# Deschloroclozapine exhibits an exquisite agonistic effect at lower concentration compared to clozapine-N-oxide in hM3Dq expressing chemogenetically modified rats

**DOI:** 10.3389/fnins.2023.1301515

**Published:** 2023-11-30

**Authors:** Makiko Shimizu, Mitsuhiro Yoshimura, Kazuhiko Baba, Naofumi Ikeda, Yuki Nonaka, Takashi Maruyama, Tatsushi Onaka, Yoichi Ueta

**Affiliations:** ^1^Department of Physiology, School of Medicine, University of Occupational and Environmental Health, Kitakyushu, Japan; ^2^Department of Orthopaedic Surgery, School of Medicine, University of Occupational and Environmental Health, Kitakyushu, Japan; ^3^Division of Brain and Neurophysiology, Department of Physiology, Jichi Medical University, Shimotsuke, Japan

**Keywords:** deschloroclozapine, clozapine-N-oxide, DREADDs, oxytocin, central nervous system, Fos, pain

## Abstract

**Introduction:**

Within the realm of chemogenetics, a particular form of agonists targeting designer receptors exclusively activated by designer drugs (DREADDs) has emerged. Deschloroclozapine (DCZ), a recently introduced DREADDs agonist, demonstrates remarkable potency in activating targeted neurons at a lower dosage compared to clozapine-N-oxide (CNO).

**Methods:**

We conducted a comparative analysis of the effects of subcutaneously administered CNO (1 mg/kg) and DCZ (0.1 mg/kg) in our transgenic rats expressing hM3Dq and mCherry exclusively in oxytocin (OXT) neurons.

**Results and Discussion:**

Notably, DCZ exhibited a swift and robust elevation of serum OXT, surpassing the effects of CNO, with a significant increase in the area under the curve (AUC) up to 3 hours post-administration. Comprehensive assessment of brain neuronal activity, using Fos as an indicator, revealed comparable effects between CNO and DCZ. Additionally, in a neuropathic pain model, both CNO and DCZ increased the mechanical nociceptive and thermal thresholds; however, the DCZ-treated group exhibited a significantly accelerated onset of the effects, aligning harmoniously with the observed alterations in serum OXT concentration following DCZ administration. These findings emphasize the remarkable efficacy of DCZ in rats, suggesting its equivalent or potentially superior performance to CNO at considerably lower dosages, thus positioning it as a promising contender among DREADDs agonists.

## 1 Introduction

The advent of chemogenetics has brought about a paradigm shift in our comprehension of neuron-specific functions, particularly in the field of behavioral research, empowering us to precisely manipulate designated neurons (Armbruster et al., 2007; Urban and Roth, 2015; Roth, 2016). The employment of agonists, such as clozapine-N-oxide (CNO), has facilitated meticulous modulation of neuronal activation, offering versatility in investigating diverse neuronal populations. Nonetheless, lingering concerns revolve around potential off-target effects and following adverse reactions associated with these pharmacological agents (Lin et al., 1996; MacLaren et al., 2016; Bærentzen et al., 2019).

CNO, a frequently utilized agonist for designer receptors exclusively activated by designer drugs (DREADDs), has been observed to possess affinity for neuronal receptors in the central nervous system (CNS) beyond its intended DREADDs targets (Bærentzen et al., 2019). Furthermore, the formation of metabolites, such as clozapine, from CNO introduces the possibility of distinct physiological roles separate from CNO *per se* (Gomez et al., 2017; Manvich et al., 2018; Jendryka et al., 2019). While the effects of CNO depend on the expression patterns of DREADDs genes and off-target effects, the potential adverse impact on animal behavior cannot be entirely disregarded, with reports of sedative effects at high doses (Padovan-Hernandez and Knackstedt, 2018).

Considering these apprehensions, deschloroclozapine (DCZ) has emerged as a promising alternative agonist for DREADDs (Nagai et al., 2020). DCZ, a metabolite of clozapine metabolized by the prominent enzyme cytochrome P450 (Pirmohamed et al., 1995), has garnered attention through ambitious investigations showcasing its comparable effects to CNO (1 mg/kg) at a mere one-tenth of the dosage (0.1 mg/kg) (Nagai et al., 2020). These characteristics of DCZ imply its suitability as a more precise DREADDs agonist, minimizing undesired interactions with non-target receptors and facilitating the discernment of specific neuronal effects. However, given that DCZ still exhibits some affinity for receptors such as dopamine D1 receptors and serotonin (5-HT) 1A receptors (Nagai et al., 2020), prudence should be exercised drawing conclusions from the experimental results, even if DREADDs can be activated using lower doses.

In our previous research, we employed CNO (1 mg/kg) to investigate the analgesic and anti-inflammatory properties of endogenous oxytocin (OXT) using OXT-hM3Dq-mCherry transgenic rats, which express hM3Dq and mCherry specifically in OXT neurons (Nishimura et al., 2022). Although DCZ has shown promise in previous studies, most of them have been conducted in mice or monkeys (Nagai et al., 2020; Matsumura et al., 2022; Perez et al., 2022). Hence, our objective was to assess the effectiveness of DCZ in our transgenic rat model. While the choice of the most appropriate animal model should be determined by specific research goals, rats possess several noteworthy advantages for scientific investigations. These advantages encompass their moderate size, ease of handling, physiological similarities to humans, potential for genetic tool utilization, suitability for neurobehavioral studies, and adherence to regulatory requirements. By exerting comparable effects to CNO at a fraction of the dosage and mitigating off-target receptor binding, the experimental protocols applied in our DREADDs rat model would be further refined and enhanced. Furthermore, considering the comparable costs of CNO and DCZ at equivalent dosages, the affordability of DCZ serves as an additional incentive for its application.

Within the scope of this research, we administered CNO (1 mg/kg) or DCZ (0.1 mg/kg) to our OXT-hM3Dq-mCherry transgenic rats, comparing OXT blood concentrations, comprehensive brain neuronal activity employing Fos protein analysis, and analgesic behavior following administration. The results revealed that even at a low dose of 0.1 mg/kg, DCZ effectively activated DREADDs and demonstrated equivalent or even superior efficacy compared to CNO (1 mg/kg). These findings have significant implications for the utilization of DCZ, not only in mice and monkeys but also in rats, including our OXT-hM3Dq-mCherry transgenic rat model. Additionally, we were able to identify the specific brain nuclei that were activated through chemogenetic stimulation of OXT, thereby incorporating these critical findings into the framework of our extensive discussions.

## 2 Materials and methods

### 2.1 Animals

Adult male OXT-hM3Dq-mCherry transgenic rats were group-housed (*n* = 3 per cage) under standard experimental conditions (temperature 23–25°C, 12/12-h light/dark cycle, lights on at 7.00 am) with *ad libitum* access to standard rat chow and tap water. All rats were handled for 7 days prior to the experiment to acclimatize the experimental condition. All experimental procedures strictly adhered to the Ethical Guidelines for the Use and Care of Laboratory Animals issued by the Japanese Physiological Society and were approved by the Ethics Committee for Animal Husbandry Experiments of the University of Occupational and Environmental Health (UOEH), Japan (Approval Number: AE21-008).

### 2.2 Drugs

Dimethyl sulfoxide (DMSO) (NACALAI TESQUE, INC., Kyoto, Japan) was diluted in physiological saline (Otsuka Pharmaceutical Co., Ltd., Tokyo, Japan). A 1% DMSO saline solution was prepared and used as the vehicle. CNO (Sigma-Aldrich Japan Co. LLC., Tokyo, Japan) was dissolved in the vehicle to a concentration of 1 mg/mL. DCZ (MedChemExpress LLC, NJ, USA) was initially dissolved in DMSO to achieve a concentration of 10 mg/mL and then further diluted in saline to a concentration of 0.1 mg/mL. The dosages of CNO and DCZ were determined based on previous studies (Nagai et al., 2020; Nishimura et al., 2022). All solutions were adjusted to ensure a final concentration of 1% DMSO.

### 2.3 Brain tissue preparation

After 120-min following subcutaneous (s.c.) injections of vehicle, CNO, or DCZ (*n* = 6 per group), rats were deeply anesthetized by s.c. injection of a combination of three types of anesthetic agents (medetomidine 0.3 mg/kg, midazolam 4.0 mg/kg, combined with butorphanol 5.0 mg/kg) and underwent transcardial perfusion with 0.1 M phosphate buffer (PB) (pH 7.4) followed by a fixative solution of 4% paraformaldehyde (PFA) dissolved in 0.1 M PB. The brains were carefully removed and post-fixed in 4% PFA for additional 48 h at 4°C, and subsequently cryoprotected by dehydration in 30% sucrose in 0.1 M PB for 48 h at 4°C. The fixed brain tissues were then sectioned into 40 μm-thick slices using a microtome (REM-700, Yamato Kohki Industrial Co. Ltd., Tokyo, Japan).

### 2.4 Fluorescence immunohistochemistry (FIHC)

The brain sections obtained from the microtome were rinsed twice with 0.1 M phosphate-buffered saline (PBS) and then washed in 0.1 M PBS (pH 7.4) containing 0.3% Triton X-100 (PBST). The sections were treated with 0.5% skimmed milk dissolved in 0.1 M PBST for 1 h to reduce non-specific binding of primary antibodies. Subsequently, the sections were incubated with a primary antibody solution (anti-c-Fos antibody raised in rabbit, Santa Cruz Biotechnology, TX, USA; 1:400 in 0.1 M PBST) for 24 h at 4°C. After the incubation, the floating sections were washed twice with 0.1 M PBST and then incubated with a secondary antibody solution (Alexa Fluor 488 donkey anti-rabbit IgG, Abcam, Cambridge, UK; 1:1,000 in 0.1 M PBST) at room temperature for 2 h. The sections were then washed twice with 0.1 M PBS, mounted on glass slides, and coverslipped using Vectashield^®^ (Vector Laboratories Co. Ltd., CA, USA).

### 2.5 Fos expression in OXT-hM3Dq-mCherry neurons

The regions of interest, the supraoptic nucleus (SON) and paraventricular nucleus (PVN), were identified based on the coordinates provided in the rat brain atlas (Paxinos and Watson, 1998). The PVN was anatomically subdivided into magnocellular PVN (mPVN) and parvocellular PVN (pPVN). We counted the number of OXT-hM3Dq-mCherry neurons and OXT-hM3Dq-mCherry neurons expressing Fos using images obtained from an All-In-One microscope (BZ-800, Keyence, Osaka, Japan). The analysis involved the quantification of Fos-positive neurons (round-shaped and green-colored nuclei), OXT-hM3Dq-mCherry-positive neurons (magenta cytoplasmic neurons), and double-positive neurons (magenta cytoplasmic neurons with green nuclei in merged images). To ensure unbiased results, cell counting was independently performed by two researchers. Number of neurons were counted in each of the three cross-sections (including six nuclei on both sides per rat), and the averaged results were obtained. Fos induction (%) was calculated as [(number of OXT-hM3Dq-mCherry neurons expressing Fos) / (number of OXT-hM3Dq-mCherry neurons)] × 100.

### 2.6 Serum OXT concentration (radioimmunoassay (RIA))

At 0, 10, 30, 60, 120, and 180 min following s.c. injections of vehicle, CNO, or DCZ, rats were decapitated without being anesthetized to exclude the impact of anesthesia-induced OXT fluctuations. A total of 89 rats, with 4–5 rats per group at each time point, were used in this experiment. Trunk blood samples were collected into chilled reaction tubes (Greiner Bio-One Co. Ltd., Kremsmuenster, Austria). The blood samples were then centrifuged at 4°C, 1,000 × *g* for 10 min. After centrifugation, a 1 mL of each serum sample was collected, and the serum OXT concentration was determined using the RIA with specific anti-OXT serum. RIAs for OXT were performed by use of an anti-OXT antibody developed and characterized in our laboratory. The standard curve of OXT was linear between 0.4 and 12.5 pg per tube. All RIAs were run at two different dilutions in duplicate.

### 2.7 3,3′-Diaminobenzidine (DAB) immunohistochemistry

The brain sections, cut by a microtome, were subjected to the following steps. They were washed twice with 0.1 M PBS and treated with a 1% hydrogen peroxide solution in 0.1 M PBS for 1 h to deactivate endogenous peroxidase activity. The sections were treated with 0.5% skimmed milk dissolved in 0.1 M PBST for 1 h to reduce non-specific binding of primary antibodies. The sections were then incubated with the primary antibody solution (anti-c-Fos antibody raised in rabbit, Santa Cruz Biotechnology, TX, USA; 1:400) for 24 h at 4°C, followed by two washes with 0.1 M PBS. Avidin/biotin solution (VECTASTAIN ABC regent, Vector Laboratories Inc., CA, USA) diluted in 0.1 M PBST was applied for 30 min, and the sections were washed twice with 0.1 M PBST. The target antigen was stained brown by incubating the sections in DAB solution (Liquid DAB+ Substrate Chromogen System, DAKO North America Inc., CA, USA) for 90 s. After two washes with 0.1 M PBS, the sections were placed on gelatin-coated glass slides and allowed to dry completely. The slides were immersed in tap water for 30 min. To sensitize the color, the slides were immersed in a 0.05% osmium tetroxide (OsO4) solution (NACALAI TESQUE INC., Kyoto, Japan) for 3 min. They were then soaked twice in distilled water, immersed in running water for 5 min, and rinsed. Dehydration was performed by immersing the slides in 70, 80, and 90% ethanol for 1 min each, anhydrous ethanol for 3 min, and xylene for 3 min. Finally, the slides were coverslipped with Entellan^®^ new (Merck KGaA, Darmstadt, Germany).

### 2.8 Enumerating Fos expression

The examined nuclei were identified based on the coordinates provided in the rat brain atlas (Paxinos and Watson, 1998). Micrographs were captured using an ECLIPSE 50i microscope (Nikon, Tokyo, Japan) and a DIGITAL SIGHT DS-Fi1 camera (Nikon, Tokyo, Japan). The number of Fos positive nuclei, appearing as round-shaped and dark brown-colored structures, was manually counted in two sections, encompassing four nuclei (including both left and right sides per rat), and the results were averaged. To ensure unbiased results, the counts were independently performed by two different researchers. The locations of Fos expression in representative sections were mapped onto templates obtained from the rat brain atlas (Paxinos and Watson, 1998).

### 2.9 Seltzer model

After inducing deep anesthesia with a s.c. injection of a combination of three types of anesthetic agents (0.3 mg/kg of medetomidine, 4.0 mg/kg of midazolam, and 5.0 mg/kg of butorphanol), the right sciatic nerve was exposed, and a 6–0 silk suture was used to ligate approximately one-third to half of its diameter, as described previously (Seltzer et al., 1990). Rats that exhibited drop-foot as a result of the procedure were excluded from the analysis.

### 2.10 von Frey and hot plate tests

Prior to testing, the rats were placed in a device and allowed to acclimate to an acrylic cage with an elevated mesh floor for at least 30 min. The mechanical sensitivity was evaluated using the manual von Frey test, which involved the use of calibrated von Frey filaments (North Coast Medical, Gilroy, CA, USA). The filaments ranged in force from 0.25 to 20.0 g were applied to the plantar surface of the ipsilateral foot in an ascending stimulus manner (Shir et al., 2001). The mechanical nociceptive threshold, defined as the weakest force (in grams) that induced a paw withdrawal response, was measured. Five stimuli were applied over a three-second interval, and the average values at each time point were calculated for each rat.

To assess heat sensitivity, a hot plate test was conducted. Rats were gently placed on a heated plate set at a temperature of 52.5°C, and the latency of nocifensive behaviors such as licking, lifting, shaking, or jumping was measured. The mean latency value was calculated and analyzed (Yamamoto et al., 2002). To ensure unbiased results, the measurements were independently performed by two different researchers.

### 2.11 Rat grimace scale (RGS)

The RGS is a widely-used behavioral assessment tool employed for evaluating pain in laboratory rats (Sotocinal et al., 2011). This scale encompasses specific facial features, including orbital tightening, nose and cheek bulges, ear changes, and whisker position. Scores are assigned to each feature based on their presence or intensity, with a score of 0 indicating no pain, a score of 2 indicating the presence of pain, and a score of 1 representing an intermediate level between the two. Pain assessments using the RGS were conducted at 0, 10, 30, 60, 120, and 180 min after the s.c. injections of vehicle, CNO, or DCZ. The individual scores were summed at each time point, and the average score was calculated. To ensure unbiased results, the scoring was independently performed by three different researchers.

### 2.12 Statistical analysis

All data underwent normality testing using D’Agnostino & Pearson, Anderson-Darling, Shapiro–Wilk and Kolmogorov–Smirnov tests, and were found to pass the normality test (alpha = 0.05) in at least one test. Subsequently, one-way ANOVA was employed for comparisons of Fos expression, while two-way ANOVA with repeated measures was used for serum concentration of OXT, von Frey and hot plate tests, and RGS, with time and treatment as the primary factors. Bonferroni multiple comparison post-tests were conducted to identify significant one-to-one differences when significance was observed by one-way ANOVA or two-way ANOVA. The level of significance was set at *p* < 0.05. Individual data point and/or mean ± SD were visually represented in all cases.

## 3 Results

### 3.1 DCZ elicits comparable Fos expression to CNO in OXT-hM3Dq-mCherry neurons within the SON and PVN

**Figure 1:**
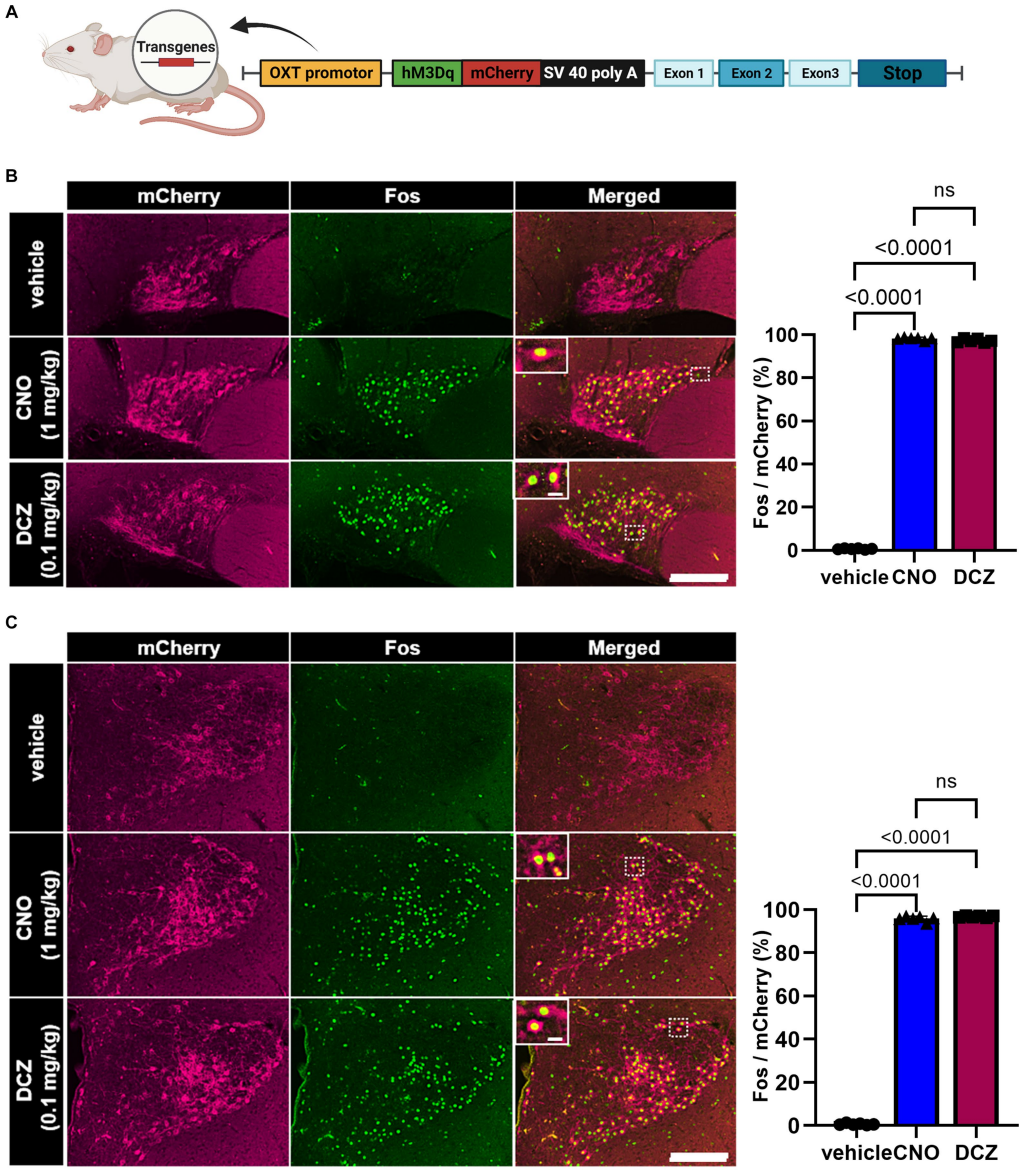
DCZ elicits comparable Fos expression to CNO in OXT-hM3Dq-mCherry neurons within the supraoptic nucleus (SON) and paraventricular nucleus (PVN). Schematic illustration of a chimeric OXT-hM3Dq-mCherry bacterial artificial chromosome (BAC) clone transgene construct **(A)**. Fos expression in OXT-hM3Dq-mCherry neurons was compared in the SON **(B)** and paraventricular nucleus (PVN) **(C)** at 120 min after subcutaneous injection of vehicle, CNO (1 mg/kg), or DCZ (0.1 mg/kg) (*n* = 6 each). Magnified images focused on the areas framed by white dotted lines. The graph represents the percentage of Fos expression in OXT-hM3Dq-mCherry neurons. Scale bars, 200 μm. Scale bars in magnified images, 10 μm. Data are presented as mean ± SD, with individual values. Figure is created with BioRender.com.

A chimeric OXT-hM3Dq-mCherry bacterial artificial chromosome (BAC) clone transgene was constructed (Figure 1A). hM3Dq-mCherry was exclusively expressed under the OXT promoter in the transgenic rats (Nishimura et al., 2022). We conducted a comparative analysis of Fos expression, a marker for neuronal activity, in OXT-hM3Dq-mCherry neurons located in the SON and PVN. Notably, both CNO and DCZ elicited significant induction of Fos in OXT-hM3Dq-mCherry neurons in both the SON [*F*(2, 15) = 44,429, *p* < 0.0001; vehicle, 0.7 ± 0.3%; CNO, 98.3 ± 0.7%; DCZ, 97.3 ± 0.9%] (Figure 1B) and PVN [*F*(2, 15) = 32,011, *p* < 0.0001; vehicle, 0.6 ± 0.4%; CNO, 95.8 ± 1.2%; DCZ, 96.8 ± 0.4%] (Figure 1C). Importantly, no significant difference was observed between the CNO and DCZ groups (SON, *p* = 0.0662; PVN, *p* = 0.1515). The PVN was further divided into mPVN and pPVN (Supplementary Figure 1A). In the vehicle, CNO, and DCZ groups, both mPVN and pPVN displayed a similar number of mCherry-positive neurons (mPVN, vehicle, 92.9 ± 13.2; CNO, 93.2 ± 12.5; DCZ, 105.6 ± 16.1; pPVN, vehicle 9.0 ± 2.5; CNO, 10.7 ± 1.4; DCZ, 12.3 ± 1.8) [mPVN, *F*(2, 15) = 1.600, *p* = 0.2344; pPVN, *F*(2, 15) = 2.346, *p* = 0.1298] (Supplementary Figure 1B). Furthermore, both mPVN and pPVN exhibited a robust induction of Fos expression following administration of CNO and DCZ (mPVN, vehicle, 0.6 ± 0.4%; CNO, 95.9 ± 1.4%; DCZ, 97.1 ± 0.4%; pPVN, vehicle, 1.1 ± 1.7%; CNO, 93.1 ± 3.4%; DCZ, 94.5 ± 2.1%) [mPVN, *F*(2, 15) = 24,447, *p* < 0.0001; pPVN, *F*(2, 15) = 2,794, *p* < 0.0001] (Supplementary Figure 1C).

### 3.2 DCZ exhibits a swift and robust elevation of serum OXT levels, surpassing the effects of CNO

**Figure 2:**
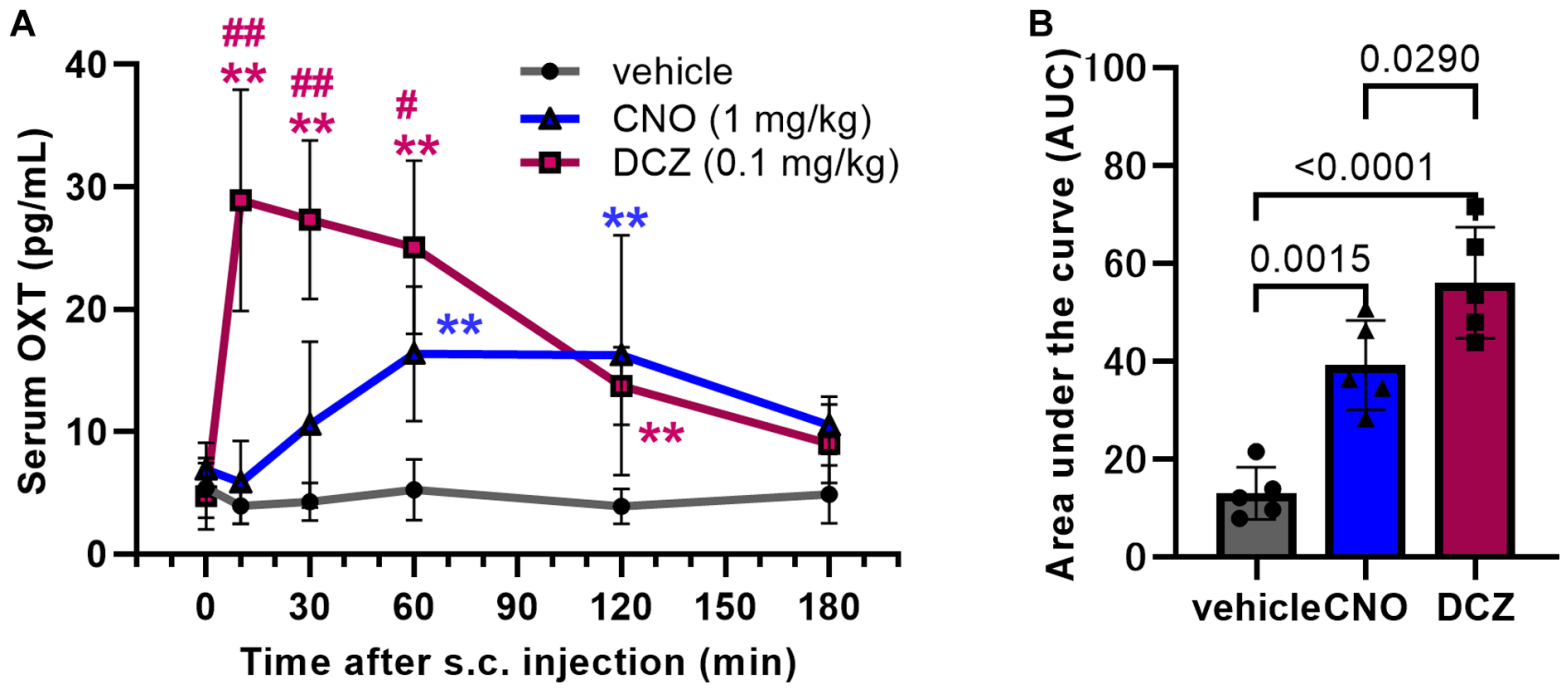
DCZ exhibits a swift and robust elevation of serum oxytocin (OXT) levels, surpassing the effects of CNO. Serum OXT concentrations were measured at 0, 10, 30, 60, 120, and 180 min following s.c. injection of vehicle, CNO (1 mg/kg), or DCZ (0.1 mg/kg) (*n* = 4–5 in each group at each time point) **(A)**. Area under the curve (AUC) of serum OXT concentration until 180 min after the s.c. injection **(B)**. Significance levels are denoted as **p < 0.01 vs. vehicle at the corresponding time point, and ^#^*p* < 0.05, ^##^*p* < 0.01 vs. CNO at the corresponding time point. Data are presented as mean ± SD, with individual values in the AUC.

Serum OXT concentration was measured using RIA at 0, 10, 30, 60, 120, and 180 min following s.c. injections of vehicle, CNO, or DCZ (Figure 2A). A two-way ANOVA with treatment and time as the main factors showed both main effects were significant [treatment, *F*(2, 71) = 56.95, *p* < 0.0001; time, *F*(5, 71) = 8.780, *p* < 0.0001], with significant interaction [*F*(10, 71) = 8.9044, *p* < 0.0001]. In the vehicle-treated rats, the concentration remained unchanged at all time points (0 min, 5.4 ± 2.4 pg/mL, 10 min, 3.9 ± 1.5 pg/mL, 30 min, 4.3 ± 1.5 pg/mL, 60 min, 5.2 ± 2.5 pg/mL, 120 min, 3.9 ± 1.4 pg/mL; 180 min, 4.9 ± 2.4 pg/mL), indicating minimal stress induced by injection and handling of the rats. In the CNO group, there was no significant increase in serum OXT concentration at 10 and 30 min, but a significant increase was observed at 60 (*p* = 0.0017) and 120 min (*p* = 0.0004) (0 min, 6.9 ± 2.2 pg/mL, 10 min, 5.9 ± 3.4 pg/mL, 30 min, 10.6 ± 6.8 pg/mL, 60 min, 16.4 ± 5.5 pg/mL, 120 min, 16.2 ± 9.8 pg/mL; 180 min, 10.5 ± 2.4 pg/mL). In the DCZ group, the serum OXT concentration was rapidly increased after s.c. administration, reaching its peak at 10 min and remaining significantly elevated until 120 min (10 min, *p* < 0.0001, 30 min, *p* < 0.0001, 60 min, *p* < 0.0001, 120 min, *p* = 0.0064), then returned comparable to the vehicle group at 180 min (0 min, 4.7 ± 2.7 pg/mL, 10 min, 28.9 ± 9.0 pg/mL, 30 min, 27.3 ± 6.5 pg/mL, 60 min, 25.1 ± 7.1 pg/mL, 120 min, 13.7 ± 3.2 pg/mL; 180 min, 9.0 ± 3.2 pg/mL).

To estimate the total release amount up to 3 h after the injections, the area under the curve (AUC) of the serum OXT concentration was calculated for each group. Both CNO and DCZ showed a significant increase compared to vehicle, with DCZ exhibiting a further increase compared to CNO [*F*(2, 12) = 29.38, *p* < 0.0001; vehicle vs. CNO, *p* = 0.0015; vehicle vs. DCZ, *p* < 0.0001; CNO vs. DCZ, *p* = 0.0290] (vehicle, 13.0 ± 5.3; CNO, 39.2 ± 9.1; DCZ, 56.0 ± 11.3) (Figure 2B). However, measurements at additional time points would be necessary for more accurate estimation of OXT release. In wild-type rats, neither CNO nor DCZ led to an increase in serum OXT concentration (Supplementary Figure 2). These findings confirm that the increase in OXT levels is not a direct consequence of CNO or DCZ themselves, but rather attributed to the effects mediated through hM3Dq.

### 3.3 CNO and DCZ induced comparable Fos expression in the median preoptic nucleus (MnPO) within the circumventricular organs (CVOs)

**Figure 3:**
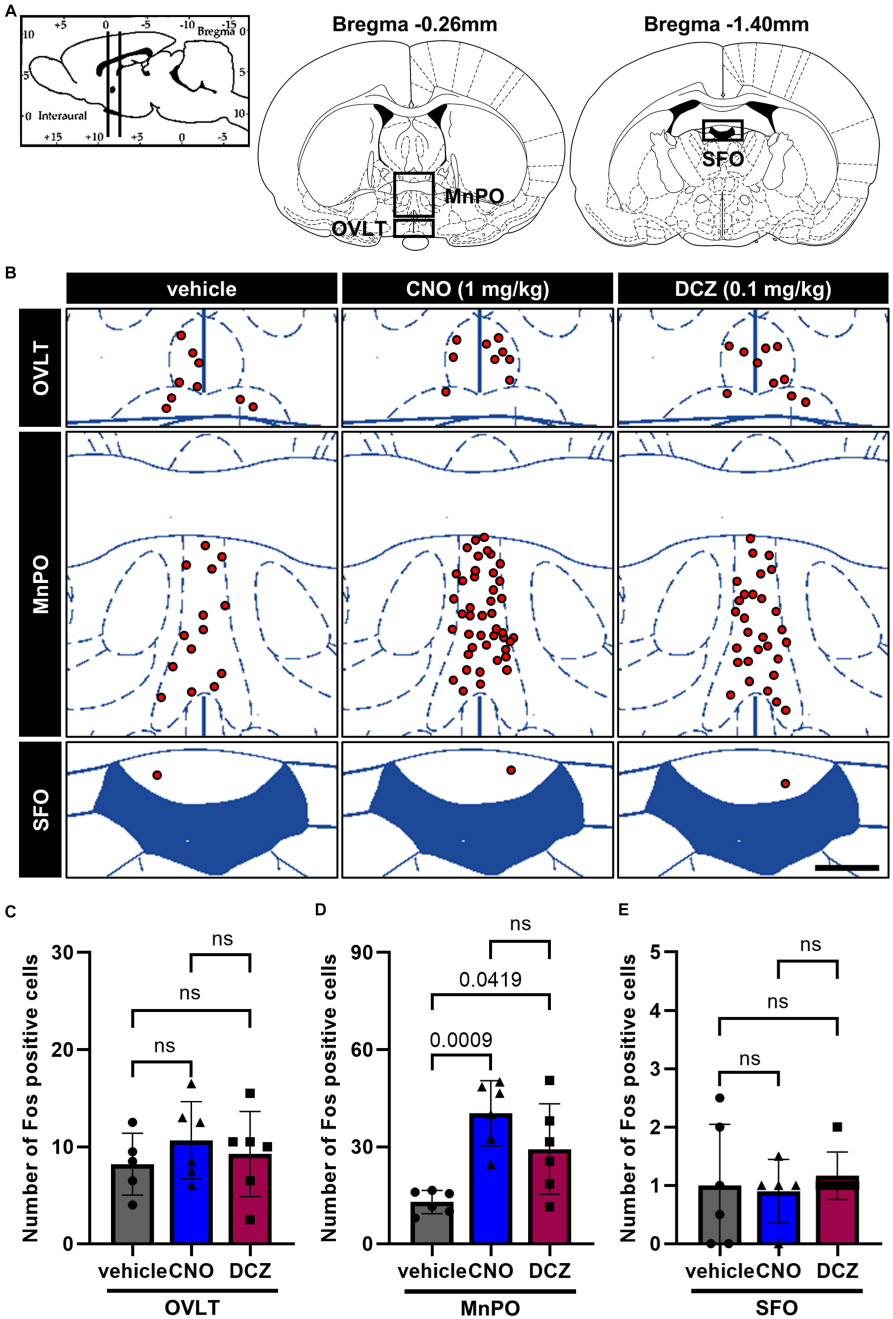
Induction of Fos in the median preoptic nucleus (MnPO) with comparable effects of CNO and DCZ. Schematic illustration of the circumventricular organs (CVOs), including organum vasculosum laminae terminalis (OVLT), MnPO, and subfornical organ (SFO) **(A)**. At 120 min after a s.c. injection of vehicle, CNO, or DCZ, the Fos expression was visually depicted as red dots on schematic diagrams of the OVLT, MnPO, and SFO, using representative brain sections **(B)**. Scale bar, 400 μm. Number of Fos positive neurons in the OVLT **(C)**, MnPO **(D)**, and SFO **(E)** (*n* = 6 each). Data are presented as means ± SD, with individual values. ns, *p* ≥ 0.05.

DAB-stained Fos expression in the CVOs, namely organum vasculosum laminae terminalis (OVLT), MPOA and subfornical organ (SFO) (Figure 3A), was assessed after s.c. injections of vehicle, CNO, or DCZ accompanied by functional mapping (Figure 3B). In the OVLT, the number of Fos expressions exhibited comparable levels among the groups [*F*(2, 14) = 0.5487, *p* = 0.5896; vehicle, 8.2 ± 3.2; CNO, 10.7 ± 4.0; DCZ, 9.3 ± 4.4] (Figure 3C). Conversely, both CNO and DCZ induced a significant increase in Fos expression within the MnPO compared to the vehicle [*F*(2, 14) = 11.03, *p* = 0.0011; vehicle vs. CNO, *p* = 0.0009; vehicle vs. DCZ, *p* = 0.0419; vehicle, 12.9 ± 3.6; CNO, 40.3 ± 10.1; DCZ, 29.3 ± 14.0] (Figure 3D). In the SFO, neither CNO nor DCZ demonstrated a notable increase in Fos expression [*F*(2, 14) = 0.1877, *p* = 0.8309; vehicle, 0.8 ± 1.0; CNO, 0.9 ± 0.5; DCZ, 1.2 ± 0.4] (Figure 3E). Both CNO and DCZ induced Fos expression in the MnPO, but not in the OVLT and SFO, where their effects were comparable (CNO vs. DCZ: OVLT, *p* > 0.9999; MnPO, *p* = 0.2359; SFO, *p* > 0.9999).

### 3.4 Fos is observed in the medial preoptic area (MPOA), SON, and PVN with comparable levels between the CNO and DCZ groups

**Figure 4:**
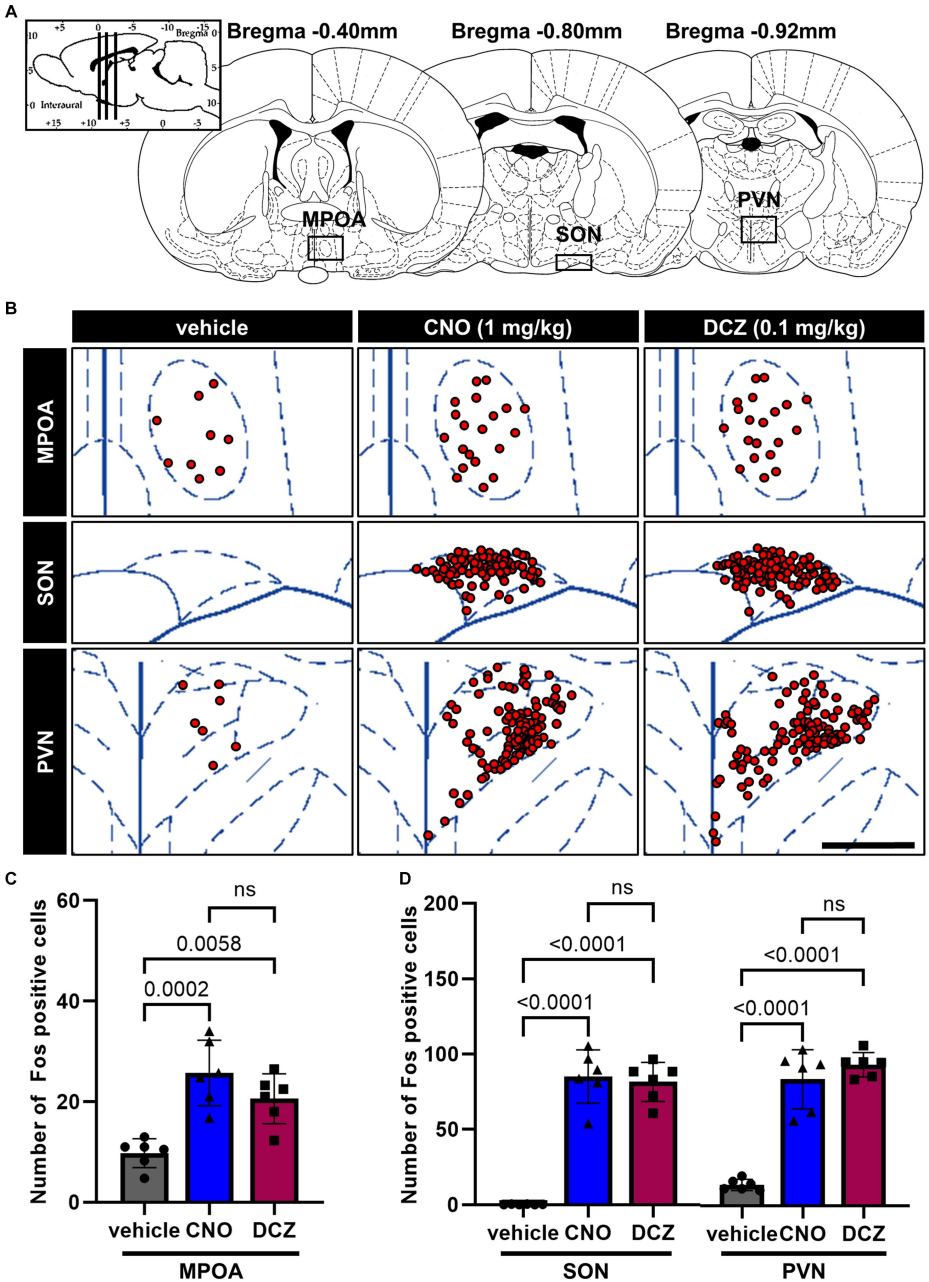
Fos is observed in the medial preoptic area (MPOA), SON, and PVN with comparable levels between the CNO and DCZ groups. Schematic illustration of the MPOA, SON and PVN **(A)**. At 120 min after a s.c. injection of vehicle, CNO, or DCZ, the Fos expression was visually depicted as red dots on schematic diagrams of the MPOA, SON, and PVN, using representative brain sections **(B)**. Scale bar, 400 μm. Number of Fos positive neurons in the MPOA **(C)**, SON, and PVN **(D)** (*n* = 6 each). Data are presented as means ± SD, with individual values. ns, *p* ≥ 0.05.

In the MPOA, SON, and PVN (Figure 4A), Fos expression was examined in DAB-stained sections after the administration of vehicle, CNO, or DCZ, accompanied by functional mapping illustrations (Figure 4B). Significant increases in Fos expression were observed in both the CNO and DCZ groups compared to the vehicle in the MPOA [*F*(2, 15) = 15.92, *p* = 0.0002; vehicle vs. CNO, *p* = 0.0002; vehicle vs. DCZ, *p* = 0.0058; vehicle, 9.8 ± 2.9; CNO, 25.7 ± 6.5; DCZ, 20.6 ± 4.9], without any statistically significance between the CNO and DCZ groups (*p* = 0.2887) (Figure 4C). Similarly, the SON and PVN exhibited significantly higher levels of Fos expression in both the CNO- and DCZ-treated groups compared to the vehicle [SON, *F*(2, 15) = 86.34, *p* < 0.0001; vehicle vs. CNO, *p* < 0.0001; vehicle vs. DCZ, *p* < 0.0001; vehicle, 0.2 ± 0.2; CNO, 85.0 ± 17.7; DCZ, 81.5 ± 12.9] [PVN, *F*(2, 15) = 73.24, *p* < 0.0001; vehicle vs. CNO, *p* < 0.0001; vehicle vs. DCZ, *p* < 0.0001; vehicle, 13.9 ± 3.7; CNO, 83.8 ± 19.6; DCZ, 93.6 ± 8.1] (Figure 4D). Notably, no significant differences were observed between the CNO- and DCZ-treated groups (SON: *p* > 0.9999; PVN: *p* = 0.5853).

### 3.5 Fos expression in the arcuate nucleus (ARC), lateral hypothalamic area (LHA), ventromedial hypothalamus (VMH), and dorsomedial hypothalamus (DMH) exhibits similar levels between the CNO and DCZ groups

**Figure 5:**
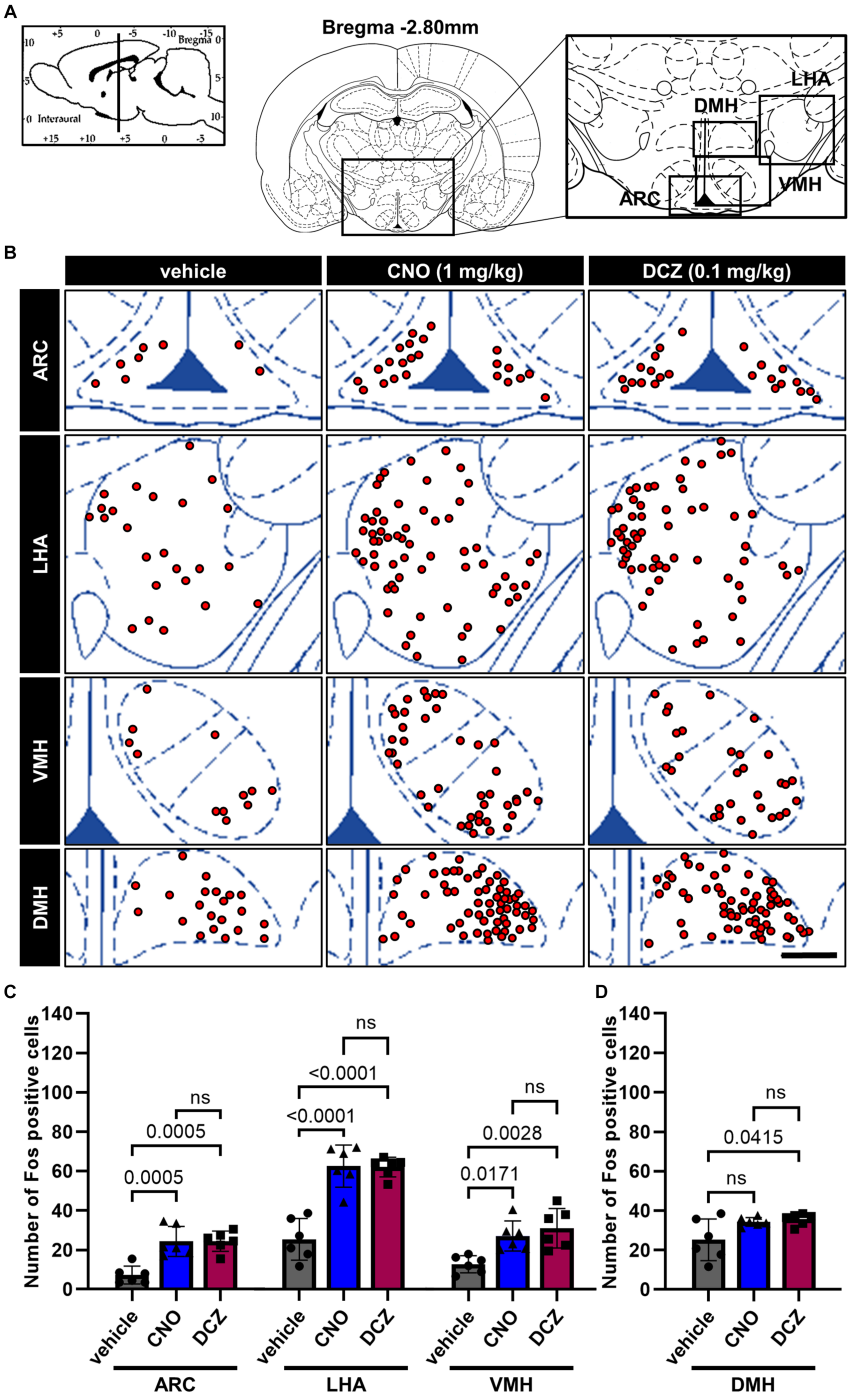
Fos expression in the arcuate nucleus (ARC), lateral hypothalamic area (LHA), ventromedial hypothalamus (VMH), and dorsomedial hypothalamus (DMH) exhibits similar levels between the CNO and DCZ groups. Schematic illustration of the ARC, LHA, DMH and VMH **(A)**. At 120 min after a s.c. injection of vehicle, CNO, or DCZ, the Fos expression was visually depicted as red dots on schematic diagrams of the ARC, LHA, DMH, and VMH, using representative brain sections **(B)**. Scale bar, 400 μm. Number of Fos positive neurons in the ARC, LHA, VMH **(C)** and DMH **(D)** (*n* = 6 each). Data are presented as means *±* SD, with individual values. ns, *p* ≥ 0.05.

Fos expression was analyzed in the ARC, LHA, VMH, and DMH (Figure 5A), following the administration of vehicle, CNO, or DCZ. Functional mapping illustrations are provided for each nucleus (Figure 5B). In the ARC, LHA, and VMH, CNO and DCZ exhibited a significant upregulation of Fos expression compared to the vehicle group [ARC, *F*(2, 15) = 16.52, *p* = 0.0002; vehicle vs. CNO, *p* = 0.0005; vehicle vs. DCZ, *p* = 0.0005; vehicle, 7.3 ± 4.6; CNO, 24.3 ± 7.7; DCZ, 24.4 ± 5.1] [LHA, *F*(2, 15) = 32.86, *p* < 0.0001; vehicle vs. CNO, *p* < 0.0001; vehicle vs. DCZ, *p* < 0.0001; vehicle, 25.2 ± 10.5; CNO, 62.3 ± 10.6; DCZ, 61.9 ± 5.0] [VMH, *F*(2, 15) = 9.353, *p* = 0.0023; vehicle vs. CNO, *p* = 0.0171; vehicle vs. DCZ, *p* = 0.0028; vehicle, 12.8 ± 4.3; CNO, 27.1 ± 7.6; DCZ, 31.1 ± 10.0]; however no significance was observed between CNO and DCZ groups (ARC, *p* > 0.9999; LHA, *p* > 0.9999; VMH, *p* > 0.9999) (Figure 5C). In the DMH [*F*(2, 15) = 4.610, *p* = 0.0275], there was no statistically significant increase in Fos expression in the CNO-treated group compared to the vehicle group (*p* = 0.0829; vehicle, 10.9 ± 5.9; CNO, 34.3 ± 2.2; DCZ, 35.6 ± 3.0). On the other hand, the DCZ-treated group showed a statistically significant increase in comparison to the vehicle group (*p* = 0.0415), without any statistical difference between the CNO and DCZ groups (*p* > 0.9999) (Figure 5D).

### 3.6 Fos is induced by chemogenetic stimulation of OXT but comparable between CNO and DCZ groups in the monoaminergic nuclei and periaqueductal gray (PAG)

**Figure 6:**
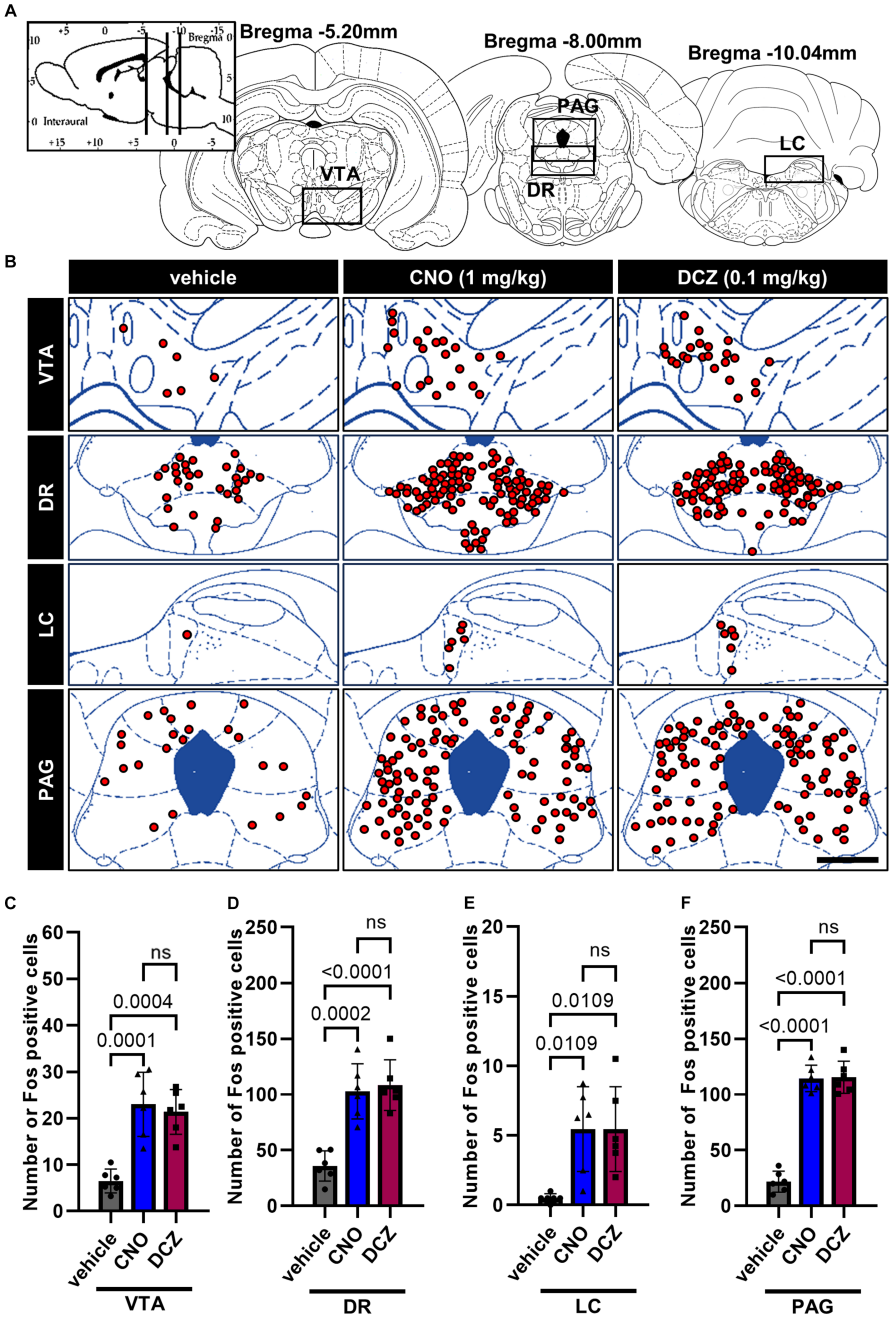
Fos is induced by chemogenetic stimulation of OXT but comparable between CNO and DCZ groups in the monoaminergic nuclei and periaqueductal gray (PAG). Schematic illustration of the ventral tegmental area (VTA), dorsal raphe nucleus (DR), locus coeruleus (LC), and PAG **(A)**. At 120 min after a s.c. injection of vehicle, CNO, or DCZ, the Fos expression was visually depicted as red dots on schematic diagrams of the VTA, DR, LC, and PAG, using representative brain sections **(B)**. Scale bar, 400 μm. Number of Fos positive neurons in VTA **(C)**, DR **(D)**, LC **(E)**, and PAG **(F)** (*n* = 6 each). Data are presented as means ± SD, with individual values. ns, *p* ≥ 0.05.

Fos expression was analyzed in the monoaminergic nuclei, including the ventral tegmental area (VTA), dorsal raphe nucleus (DR), and locus coeruleus (LC), and PAG (Figure 6A), following the administration of vehicle, CNO, or DCZ. Functional mapping illustrations are provided for each nucleus (Figure 6B). CNO and DCZ groups exhibited a significant increase in Fos expression compared to vehicle group in these nuclei [VTA, *F*(2, 15) = 19.16, *p* < 0.0001; vehicle vs. CNO, *p* = 0.0001; vehicle vs. DCZ, *p* = 0.0004; vehicle, 6.5 ± 2.5; CNO, 23.0 ± 6.9; DCZ, 21.3 ± 4.8] (Figure 6C) [DR, *F*(2, 15) = 22.27, *p* < 0.0001; vehicle vs. CNO, *p* = 0.0002; vehicle vs. DCZ, *p* < 0.0001; vehicle, 35.8 ± 13.6; CNO, 102.8 ± 24.8; DCZ, 108.4 ± 22.8] (Figure 6D) [LC, *F*(2, 15) = 7.901, *p* = 0.00045; vehicle vs. CNO, *p* = 0.0109; vehicle vs. DCZ, *p* = 0.0109; vehicle, 0.5 ± 0.3; CNO, 5.5 ± 3.0; DCZ, 5.5 ± 3.3] (Figure 6E) [PAG, *F*(2, 15) = 118.9, *p* < 0.0001; vehicle vs. CNO, *p* < 0.0001; vehicle vs. DCZ, *p* < 0.0001; vehicle, 21.8 ± 9.4; CNO, 114.4 ± 11.9; DCZ, 115.5 ± 14.4] (Figure 6F), without any significant differences between the CNO and DCZ groups (VTA, *p* > 0.9999; PAG, *p* > 0.9999; DR, *p* > 0.9999; LC, *p* > 0.9999).

### 3.7 DCZ elicits comparable Fos expression to CNO in the brainstem

**Figure 7:**
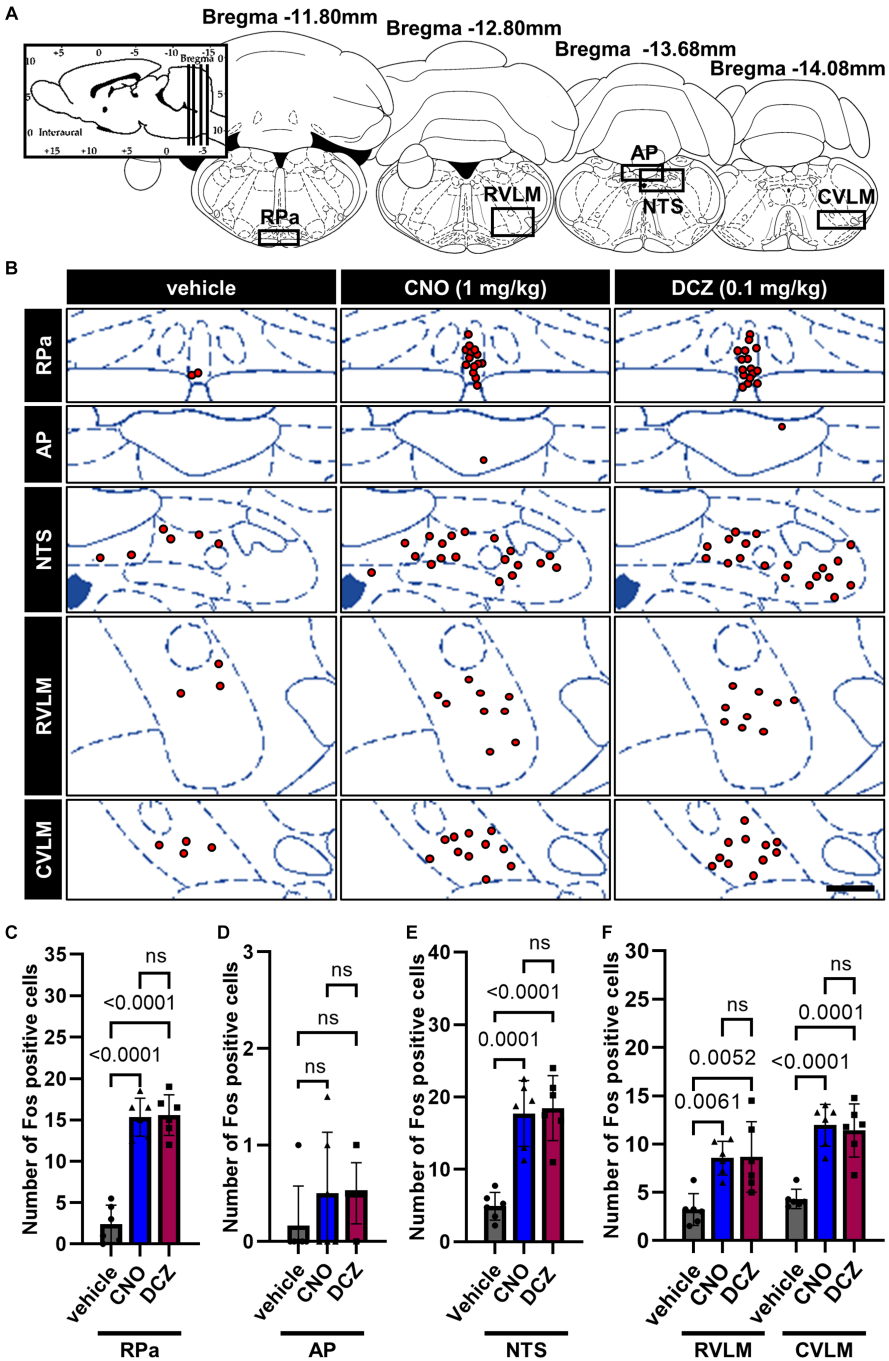
DCZ elicits comparable Fos expression to CNO in the brainstem. Schematic illustration of the raphe pallidus (RPa), area postrema (AP), nucleus tractus solitarius (NTS), rostral ventrolateral medulla (RVLM) and caudal ventrolateral medulla (CVLM) **(A)**. At 120 min after a s.c. injection of vehicle, CNO, or DCZ, the Fos expression was visually depicted as red dots on schematic diagrams of the RPa, AP, NTS, RVLM, and CVLM, using representative brain sections **(B)**. Scale bar, 400 μm. Number of Fos positive neurons in RPa **(C)**, AP **(D)**, NTS **(E)**, RVLM and CVLM **(F)** (*n* = 6 in each group). Data are presented as means ± SD, with individual values. ns, *p* ≥ 0.05.

Fos expression was assessed in the brain stem, specifically in the raphe pallidus (RPa), area postrema (AP), nucleus tractus solitarius (NTS), rostral ventrolateral medulla (RVLM) and caudal ventrolateral medulla (CVLM) (Figure 7A), following the administration of vehicle, CNO, or DCZ. Functional mapping illustrations are provided for each nucleus (Figure 7B). In the RPa, both CNO and DCZ induced a significant increase in the number of Fos expression compared to vehicle, without any statistical difference between CNO and DCZ [*F*(2, 15) = 62.05, *p* < 0.0001; vehicle vs. CNO, *p* < 0.0001; vehicle vs. DCZ, *p* < 0.0001; CNO vs. DCZ, *p* > 0.9999; vehicle, 2.4 ± 2.3; CNO, 15.3 ± 2.3; DCZ, 15.6 ± 2.5] (Figure 7C). In the AP, no discernible difference was seen in the number of Fos expression [*F*(2, 15) = 1.000, *p* = 0.3911, vehicle, 0.2 ± 0.4; CNO, 0.5 ± 0.6; DCZ, 0.5 ± 0.3] (Figure 7D). In the NTS, both CNO and DCZ exhibited a significant increase in Fos expression compared to the vehicle group. However, no statistically difference was observed between the effects of CNO and DCZ [*F*(2, 15) = 23.35, *p* < 0.0001; vehicle vs. CNO, *p* = 0.0001; vehicle vs. DCZ, *p* < 0.0001; CNO vs. DCZ, *p* > 0.9999; vehicle, 4.9 ± 0.9; CNO,17.7 ± 1.9; DCZ, 18.5 ± 1.8] (Figure 7E). Either CNO or DCZ demonstrated a significant induction of Fos expression in both the RVLM and CVLM, compared to the vehicle group, without any significant difference between the CNO and DCZ groups [RVLM, *F*(2, 15) = 9.168, *p* = 0.0025; vehicle vs. CNO, *p* = 0.0061; vehicle vs. DCZ, *p* = 0.0052, CNO vs. DCZ, *p* = 0.9959; vehicle, 3.2 ± 1.7; CNO, 8.5 ± 1.7; DCZ, 8.6 ± 3.6] [CVLM, *F*(2, 15) = 24.55, *p* < 0.0001, vehicle vs. CNO, *p* < 0.0001; vehicle vs. DCZ, *p* < 0.0001, CNO vs. DCZ, *p* > 0.9999; vehicle, 4.3 ± 1.0; CNO, 11.9 ± 2.2; DCZ, 11.3 ± 2.8] (Figure 7F). Representative images captured by a digital camera are presented as supplementary illustrations (Supplementary Figures 3–6).

### 3.8 DCZ demonstrates rapid and equivalent analgesic effects in response to mechanical stimuli compared to CNO in a neuropathic pain model

**Figure 8:**
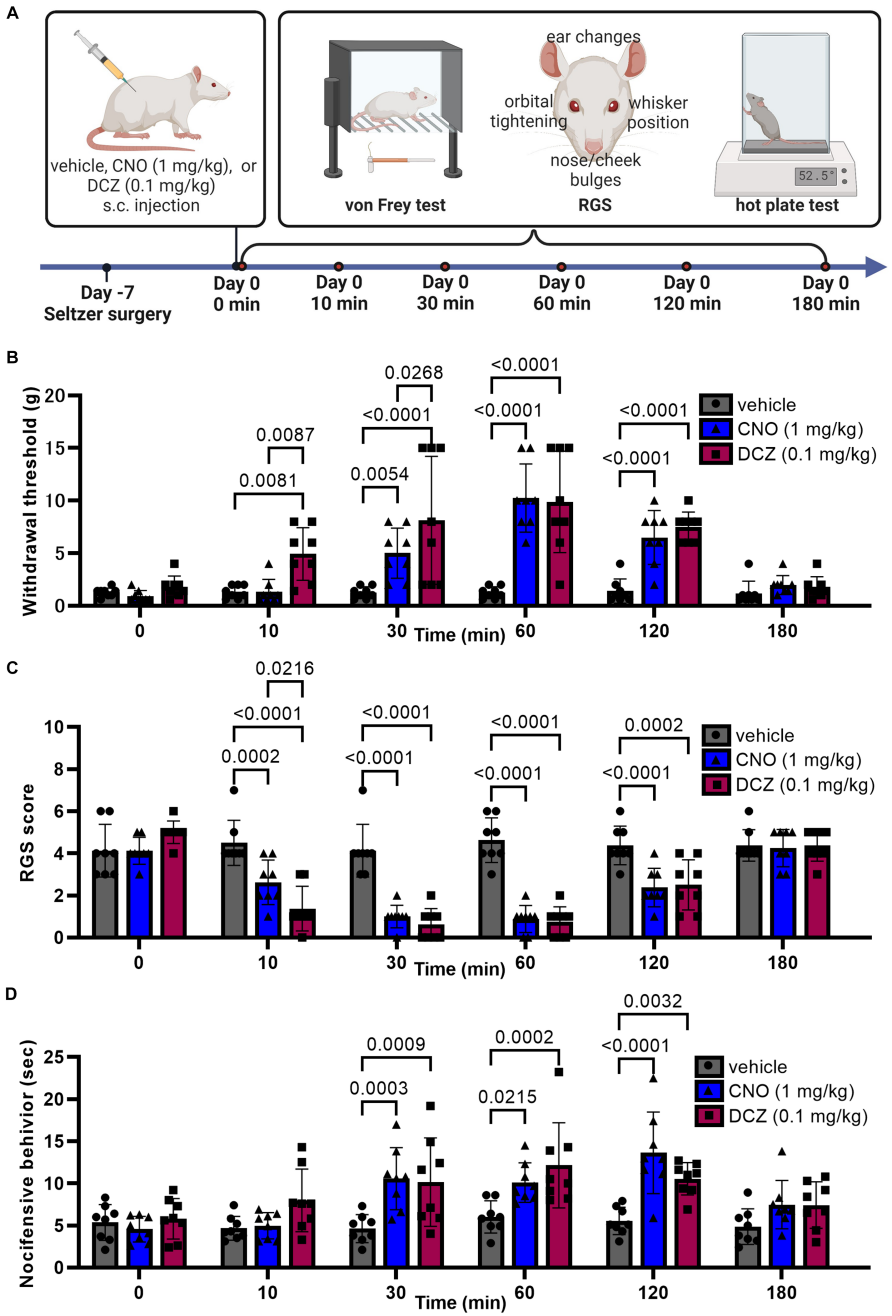
DCZ demonstrates rapid and equivalent analgesic effects in response to mechanical stimuli compared to CNO in a neuropathic pain model. Schematic illustration of experimental procedure for von Frey test, RGS, and hot plate test in the Seltzer model **(A)**. Results of the von Frey test **(B)**, RGS **(C)**, and hot plate test **(D)** after a s.c. injection of vehicle, CNO, or DCZ in the Seltzer model (*n* = 8 each). Data are represented as mean ± SD, with individual values. Figure is created with BioRender.com.

Seven days after the Seltzer surgery, von Frey test, RGS, and hot plate test were analyzed at 0, 10, 30, 60, 120, and 180 min after s.c. injection of vehicle, CNO, or DCZ (Figure 8A).

In the von Frey test (Figure 8B), a two-way ANOVA with repeated measures with treatment and time as the main factors showed both main effects were significant [treatment, *F*(2, 126) = 43.70, *p* < 0.0001; time, *F*(5, 126) = 22.72, *p* < 0.0001], with significant interaction between the two [*F*(10, 126) = 6.604, *p* < 0.0001]. The vehicle group showed no significant changes in withdrawal threshold at any time point, confirming that the injection and handling of the rats did not affect the results (0 min, 1.3 ± 0.4 g, 10 min, 1.3 ± 0.6 g, 30 min, 1.3 ± 0.6 g, 60 min, 1.3 ± 0.6 g, 120 min, 1.4 ± 1.2 g; 180 min, 1.2 ± 1.2 g). In the CNO-treated group, a significant increase in the withdrawal threshold was observed between 30, 60, and 120 min, reaching its peak at 60 min (0 min, 0.9 ± 0.5 g, 10 min, 1.4 ± 1.2 g, 30 min, 5.0 ± 2.4 g, 60 min, 10.3 ± 3.2 g, 120 min, 6.5 ± 2.6 g; 180 min, 2.0 ± 0.9 g). On the other hand, the DCZ group exhibited a rapid and significant increase at 10 min. The effect lasted for up to 120 min, similar to CNO (0 min, 1.8 ± 1.0 g, 10 min, 4.9 ± 2.5 g, 30 min, 8.1 ± 6.1 g, 60 min, 9.9 ± 4.8 g, 120 min, 7.5 ± 1.4 g; 180 min, 1.7 ± 1.0 g). Data for both groups returned to baseline levels by 180 min. Notably, there was no significant difference in the peak withdrawal threshold between the two groups.

In the RGS (Figure 8C), a two-way ANOVA with repeated measures with treatment and time as the main factors showed both main effects were significant [treatment, *F*(2, 126) = 66.59, *p* < 0.0001; time, *F*(5, 126) = 33.02, *p* < 0.0001], with significant interaction between the two [*F*(10, 126) = 10.63, *p* < 0.0001]. The vehicle group exhibited stable RGS scores (0 min, 4.1 ± 1.2, 10 min, 4.5 ± 1.1, 30 min, 4.1 ± 1.2, 60 min, 4.6 ± 1.1, 120 min, 4.4 ± 0.9; 180 min, 4.4 ± 0.7). Both CNO and DCZ groups displayed a significant decrease in RGS scores at 10, 30, 60 and 120 min compared to the vehicle group, returning to baseline at 180 min (CNO, 0 min, 4.1 ± 0.6; 10 min, 2.6 ± 1.0; 30 min, 1.0 ± 0.5; 60 min, 0.9 ± 0.6; 120 min, 2.4 ± 0.9; 180 min, 4.3 ± 0.9) (DCZ, 0 min, 5 ± 0.5; 10 min, 1.4 ± 1.1; 30 min, 0.6 ± 0.7; 60 min, 0.8 ± 0.7; 120 min, 2.5 ± 1.2; 180 min, 4.4 ± 0.7). Of note, the RGS score of the DCZ-treated group was notably lower than that of the CNO-treated group at 10 min post-injection (*p* = 0.0216). These findings are consistent with the results obtained from the von Frey test.

In the hot plate test (Figure 8D), a two-way ANOVA with repeated measures with treatment and time as the main factors demonstrated both main effects were significant [treatment, *F*(2, 126) = 23.58, *p* < 0.0001; time, *F*(5, 126) = 10.23, *p* < 0.0001], with significant interaction between the two [*F*(10, 126) = 3.086, *p* = 0.0015]. The vehicle group showed stable latency of nocifensive behavior (0 min, 5.4 ± 2.1 s, 10 min, 4.7 ± 1.4 s, 30 min, 4.6 ± 1.7 s, 60 min, 6.0 ± 1.9 s, 120 min, 5.5 ± 1.6 s; 180 min, 4.9 ± 2.1 s), whereas both the CNO and DCZ groups displayed a significant increase in such behavior at 30, 60, and 120 min. Unlike the result of von Frey test and RGS sore, there was no significant difference between the CNO and DCZ groups at any time point (CNO, 0 min, 4.6 ± 1.5 s; 10 min, 5.0 ± 1.6 s; 30 min, 10.6 ± 3.7 s; 60 min, 10.1 ± 2.3 s; 120 min, 13.6 ± 4.9 s; 180 min, 7.4 ± 2.9 s) (DCZ, 0 min, 5.8 ± 2.4 s; 10 min, 8.0 ± 3.7 s; 30 min, 10.2 ± 5.3 s; 60 min, 12.2 ± 5.1 s; 120 min, 10.5 ± 1.9 s; 180 min, 7.4 ± 2.8 s).

## 4 Discussion

Our findings indicate that, in our genetically modified rats, DCZ may possess comparable or potentially superior efficacy to CNO, despite being administered at only one-tenth of the dosage. Analysis of Fos protein suggests that both CNO and DCZ effectively stimulate neuronal activity in the brain. Additionally, DCZ exhibits a notably higher concentration of serum OXT and a faster onset of anti-nociceptive effect in a neuropathic pain model.

The PVN is anatomically constituted of two primary regions. The mPVN projects axons to the posterior pituitary, and the pPVN extends its projections to the central nervous system (Wang et al., 2022). In our genetically modified rat model, it is possible to selectively activate OXT neurons; however, site-specific activation of OXT neurons in either the mPVN or pPVN is not achievable. Given that OXT neuronal populations in each subregion may possess distinct functions, alternative methods are necessary to identify region-specific functionality.

Previous studies have suggested that CNO does not cross the blood–brain barrier (BBB), while it is believed that DCZ has the ability to penetrate the BBB (Manvich et al., 2018; Nagai et al., 2020). It is plausible that the administered CNO may undergo metabolism, resulting in the formation of clozapine or DCZ, which can subsequently bind to DREADDs. Our previous investigation has confirmed the expression of hM3Dq-mCherry in the posterior pituitary (Nishimura et al., 2022), indicating that even if CNO fails to cross the BBB, rapid release of OXT from the BBB-lacking posterior pituitary could occur if CNO effectively binds to hM3Dq. In our experiments using OXT-hM3Dq-mCherry transgenic rats, DCZ led to a faster and more pronounced release of OXT compared to CNO. The shorter duration required to observe significant effects in the pain model with DCZ, along with the corresponding changes in serum OXT concentration, support this observation. The underlying reasons for these differences, whether attributed to variations in DREADDs affinity between CNO and DCZ or the influence of metabolites, warrant further discussion. Furthermore, it is crucial to acknowledge that blood concentration solely reflects peripheral OXT levels and does not provide insights into the activation response rate of OXT neurons or the binding kinetics of CNO or DCZ to the targeted DREADDs in the brain.

There are diverse reports and perspectives regarding the half-life of CNO and DCZ (Allen et al., 2019; Oyama et al., 2022). It is possible that their administration routes can influence their respective half-lives (Ferrari et al., 2022). Based on our observations of OXT blood concentration and the pain model, it is hypothesized that both CNO and DCZ have a half-life ranging from several tens of minutes to an hour. Nevertheless, it cannot be definitively ruled out that sustained stimulation of DREADDs may induce alterations in the excitation threshold or release quantity of specific substances, making it challenging to accurately predict the half-life of CNO and DCZ based solely on serum OXT concentration. This all being said, it should be acknowledged that chemogenetics does not provide the same precise temporal resolution as optogenetics (Vlasov et al., 2018), thus determining the exact onset time of effects may be of lesser significance. When utilizing DCZ as a DREADDs agonist, however, it is important to take into account its faster onset of effects in comparison to CNO. Additionally, there are other commercially available DREADDs agonists to consider (Chen et al., 2015; Thompson et al., 2018), and their selection should be aligned with the specific goals of the research question.

The Fos protein analysis suggests that the activation of OXT neurons might be associated with the excitation of multiple nuclei, without any significant differences observed between CNO and DCZ. This suggests that DCZ exhibits comparable effects to CNO at significantly lower dose. In addition, CNO at a dose of 1 mg/kg may not raise significant concerns regarding off-target effects (Traut et al., 2023). The lack of differences between CNO and DCZ in *in vivo* Fos observations can be considered as a significant finding of this study. Both CNO and DCZ are known to have off-target effects on dopamine and 5-HT receptors (Bærentzen et al., 2019; Nagai et al., 2020). Since monoamine receptors are expressed in various brain regions and the specific activation of OXT neurons can stimulate monoamine neurons, discerning the off-target effects of DREADDs agonists in our genetically modified rats poses a formidable challenge. To investigate off-target effects in more detail, it would be necessary to test CNO and DCZ in wild-type rats, although this would deviate from the purpose of this study.

The crucial point to bear in mind is that we cannot negate the cross reaction of oxytocin through the Vasopressin 1a (V1a) receptors. V1a receptors are expressed in various parts of the brain and have affinity with OXT (Song and Albers, 2018). Thus, it was possible that the observed Fos expression in nuclei lacking OXT receptors might have been induced through the binding of OXT to V1a receptors. Future studies delving into this interaction may shed light on the responses observed in our experimental model. Additionally, it is important to note that our observations were limited to Fos protein expression and may not encompass all brain neuronal activities.

Significant Fos expression was observed in both the CNO and DCZ groups within the MnPO, a crucial component of the CVOs. The MnPO is known to express OXT receptors and is involved in various physiological functions, including thermoregulation (Maruyama et al., 2003), control of fluid and food intake (Rowland et al., 2000; Fitts et al., 2003), regulation of the cardiovascular system (Bealer, 2000), modulation of reproductive behaviors (Cera et al., 2021), and sleep regulation (Uschakov et al., 2007). The observed increase in Fos expression in this nucleus suggests the activation of OXT neurons through chemogenetic stimulation, which aligns with the reported effects of OXT (Wang et al., 2022).

In the SON and PVN, it is presumed that the majority of Fos expression occurred in OXT neurons. The MPOA has been reported to be involved in the regulation of maternal and reproductive behaviors, and it expresses OXT receptors (Gil et al., 2013). The observed Fos expression in the MPOA may potentially result from the activation of OXT neurons. The MPOA encompasses the sexually dimorphic nucleus, known as sexually dimorphic nucleus of the preoptic area (SDN-POA). Calbindin, a type of calcium-binding protein, has been utilized as a marker for SDN-POA (Tsuneoka et al., 2017). It remains unclear which neurons within the MPOA are responsible for the observed Fos expression associated with the activation of OXT neurons. Future investigations employing dual immunostaining for calbindin and Fos will be necessary to elucidate the functional properties of these activated neurons.

The ARC, LHA, VMH, and DMH include a multitude of neurons with various functions among other functions, all of which play a role in appetite regulation with distinct roles in appetite stimulation or suppression (Bouret, 2017). Furthermore, within each of these nuclei, both orexigenic and anorexigenic peptides are produced, as exemplified by the ARC. While the results of this study suggest that the activation of OXT neurons might be involved in appetite regulation, confirming the veracity of this claim and understanding its mechanisms solely on Fos protein analysis is challenging. Previous studies have suggested anorexigenic effects of OXT (Liyanagamage et al., 2023), while others have shown that chemogenetic activation of OXT does not affect food intake (Sutton et al., 2014). Further investigations are necessary to fully understand the involvement of OXT in feeding behavior. Nevertheless, the lack of significant differences in Fos protein expression between the CNO and DCZ groups implies that the effects of OXT neuron activation are comparable.

Activation of OXT neurons led to increased Fos expression in monoaminergic nuclei and PAG, which is consistent with our previous findings (Nishimura et al., 2022). Importantly, the PAG, DR, and LC are neural nuclei involved in pain control through descending pain antinociceptive systems (Moriya et al., 2019). The significant increase in Fos protein in these nuclei provides support for the observed pain relief effects in the rats with a neuropathic pain (Sun et al., 2018).

The RPa, CVLM, and RVLM are nuclei involved in the autonomic nervous system, responsible for regulating body temperature and blood pressure (Macefield and Henderson, 2019). Significant increases in Fos expression were observed in these nuclei despite the absence of reports of OXT receptor expression in these nuclei. However, it is known that monoamine receptors, including 5-HT receptors, are expressed in these nuclei (Asmundsson et al., 1997; Hornung, 2003). These suggest that the observed Fos expression in these nuclei may be indirectly influenced by the activation of monoamine neurons, rather than through a direct impact of OXT neuron activation. Since the effects of OXT on body temperature and blood pressure regulation lack a definitive consensus (Gutkowska et al., 2014; Yosten and Samson, 2014; Kohli et al., 2019; Fukushima et al., 2022), further investigations are necessary to fully understand the underlying mechanisms.

The AP primarily processes peripheral information from the systemic circulation and does not express OXT receptors (Verbalis, 1999), which explains the absence of Fos expression in this region. Morton et al. (2012) reported that peripheral administration of OXT led to an increase in Fos expression in the AP. However, supraphysiological dose of OXT were administered in this study, thus it cannot be concluded that endogenous OXT affects the AP. The NTS contains OXT receptors as well as monoamine receptors, and it is believed that OXT can activate the NTS (Ong et al., 2017). Activation of the NTS by OXT has the potential to influence autonomic regulation, stress response, appetite control, and blood pressure regulation (Karelina and Norman, 2009). Fos expression induced by OXT neuronal activation indicates that OXT possibly influence these regulations through the NTS neurons. However, considering that OXT can stimulate monoamine neurons, further investigations are warranted to elucidate whether the activation of the NTS neurons is mediated through OXT receptors or monoamine receptors.

Drawing from the findings of brain Fos expression analysis, no significant disparities were detected among the nuclei investigated between CNO (1 mg/kg) and DCZ (0.1 mg/kg). These findings suggest that even at a low dosage of DCZ (0.1 mg/kg), comparable activation of brain nuclei was observed, akin to that achieved with CNO (1 mg/kg).

In summary, the results of this study lend support to the assertive utilization of DCZ as a DREADDs agonist in rats. The integration of the DREADDs system with pluripotent stem cells and direct reprogramming techniques is increasingly being acknowledged as a valuable resource in the quest for therapeutic interventions targeting neurological and muscular disorders (Dell’Anno et al., 2014; Kawai et al., 2021). With its demonstrated efficacy and rapid, potent effects at low dose, DCZ might emerge as a promising contender in advancing translational research, offering new avenues for exploration.

## Data availability statement

The raw data supporting the conclusions of this article will be made available by the authors, without undue reservation.

## Ethics statement

The animal study was approved by Ethics Committee for Animal Husbandry Experiments of the University of Occupational and Environmental Health (Approval Number: AE21-008). The study was conducted in accordance with the local legislation and institutional requirements.

## Author contributions

MS: Data curation, Formal analysis, Investigation, Writing – original draft. MY: Conceptualization, Data curation, Formal analysis, Funding acquisition, Investigation, Supervision, Validation, Writing – original draft, Writing – review & editing. KB: Data curation, Investigation, Writing – review & editing. NI: Data curation, Investigation, Writing – review & editing. TM: Writing – review & editing. TO: Data curation, Investigation, Writing – review & editing. YU: Writing – review & editing. YN: Data curation, Formal analyis.
