# Supplementary material for: Deschloroclozapine exhibits an exquisite agonistic effect at lower concentration compared to clozapine-N-oxide in hM3Dq expressing chemogenetically modified rats
Source: Front Neurosci. 2023 Nov 30;17:1301515. doi: 10.3389/fnins.2023.1301515 (PMC10720889; doi:10.3389/fnins.2023.1301515)
Supplement: Supplementary file 1 [file Data_Sheet_1.docx]

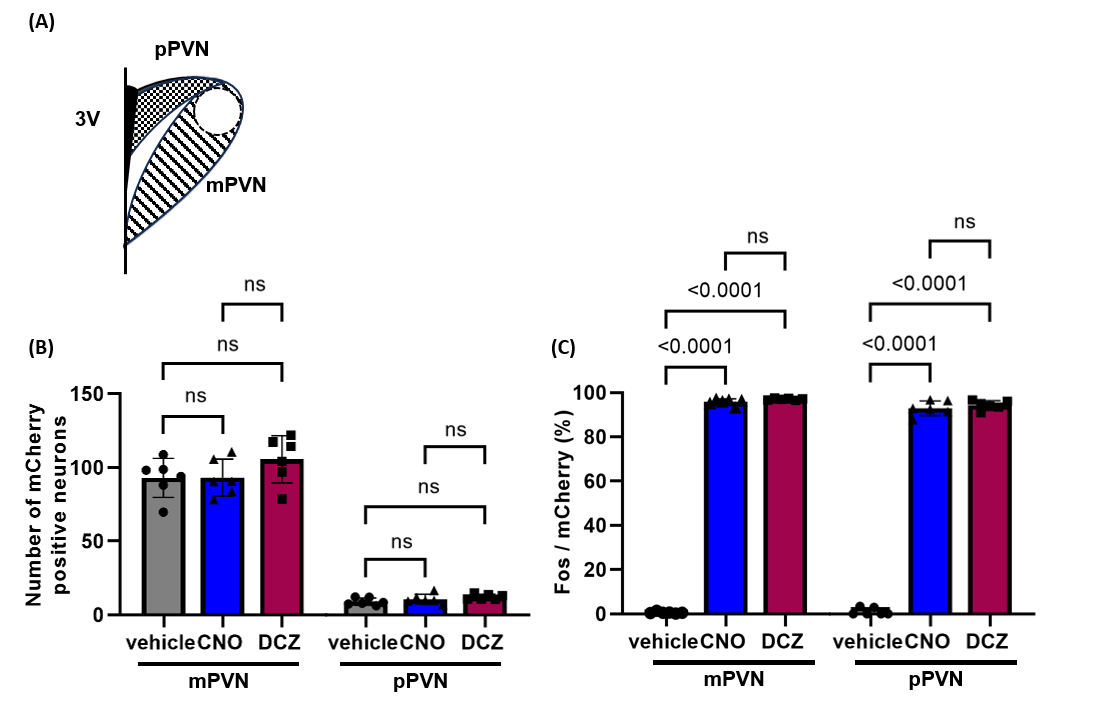


**Supplementary Figure 1. Both magnocellular PVN (mPVN) and parvocellular PVN (pPVN) OXT neurons are activated by DREADDs agonists.**

Schematic representation of the mPVN and pPVN (A). Number of mCherry-positive neurons in the mPVN and pPVN. Number of mCherry neurons are comparable among the groups (B). Fos expression in the mPVN and pPVN OXT neurons (C). Data are presented as mean ± SD, with individual values. ns, *p*≥0.05.


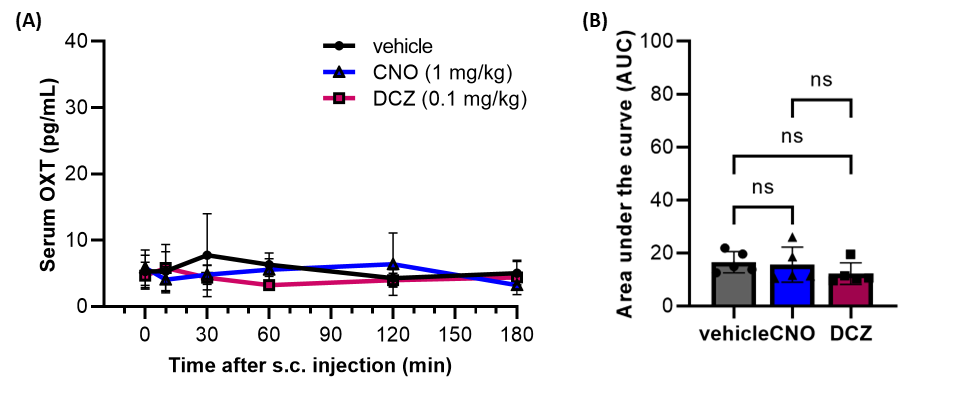


**Supplementary Figure 2. Serum oxytocin (OXT) was not affected by CNO or DCZ in adult male wild type rats.**

Serum OXT concentrations were measured by radioimmunoassay (RIA) at 0, 10, 30, 60, 120, and 180 min after a subcutaneous (s.c.) injection of vehicle, CNO (1 mg/kg), or DCZ (0.1 mg/kg) in adult male wild type rats (n=4-5 in each group at each time point) (A). Area under the curve (AUC) of serum OXT concentration until 180 min after the s.c. injection (B). Data are presented as mean ± SD, with individual values in the AUC. ns, *p*≥0.05.


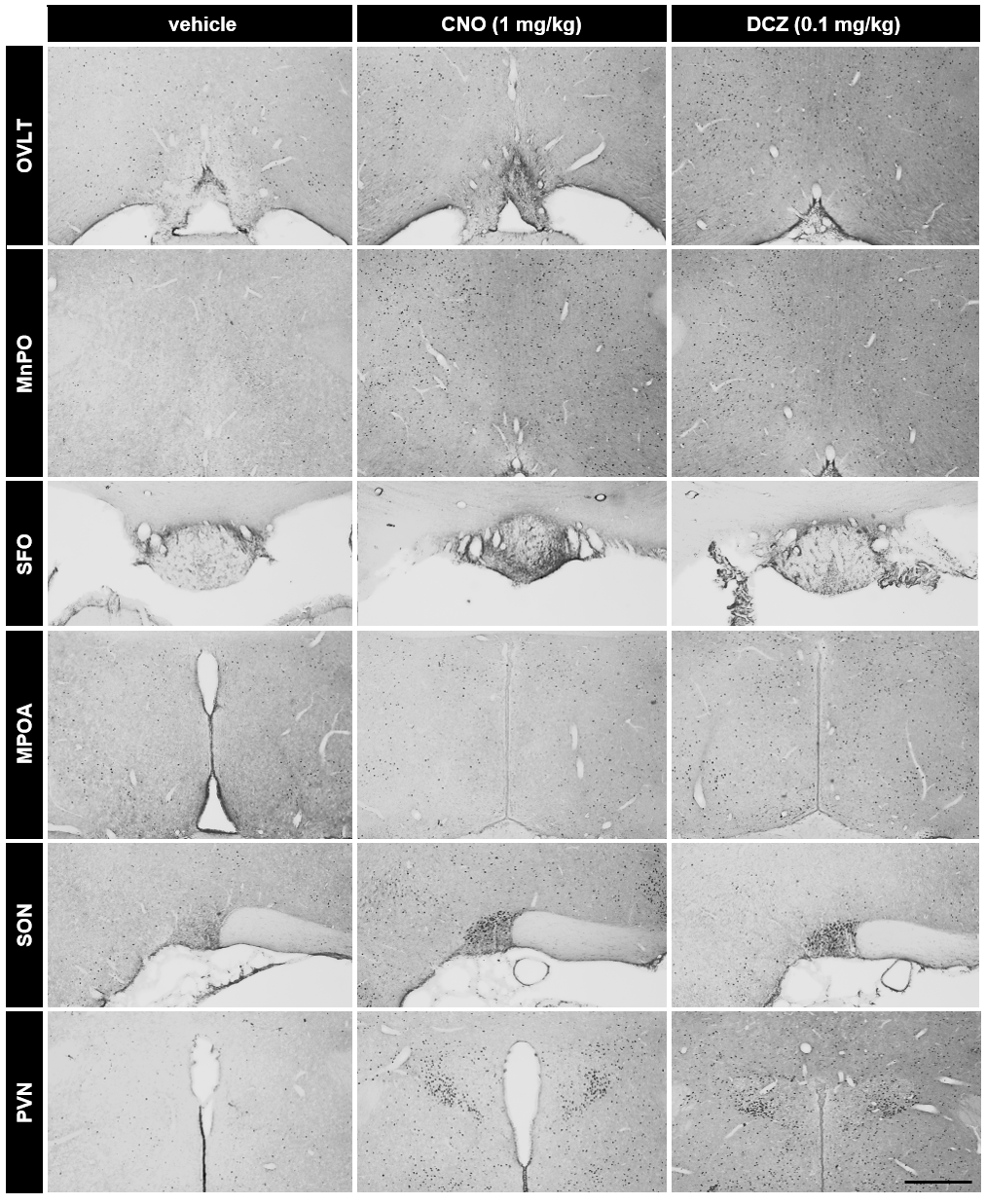


**Supplementary Figure 3. Representative images of DAB immunostaining for Fos in the organum vasculosum laminae terminalis (OVLT), median preoptic nucleus (MnPO), and subfornical organ (SFO), medial preoptic area (MPOA), supraoptic nucleus (SON), and paraventricular nucleus (PVN).**

The images are obtained at 120 min after s.c. injections of vehicle, CNO, or DCZ. Fos-positive neurons are stained by dark brown. Images are converted to black and white to improve visibility. Scale bar, 400 µm.


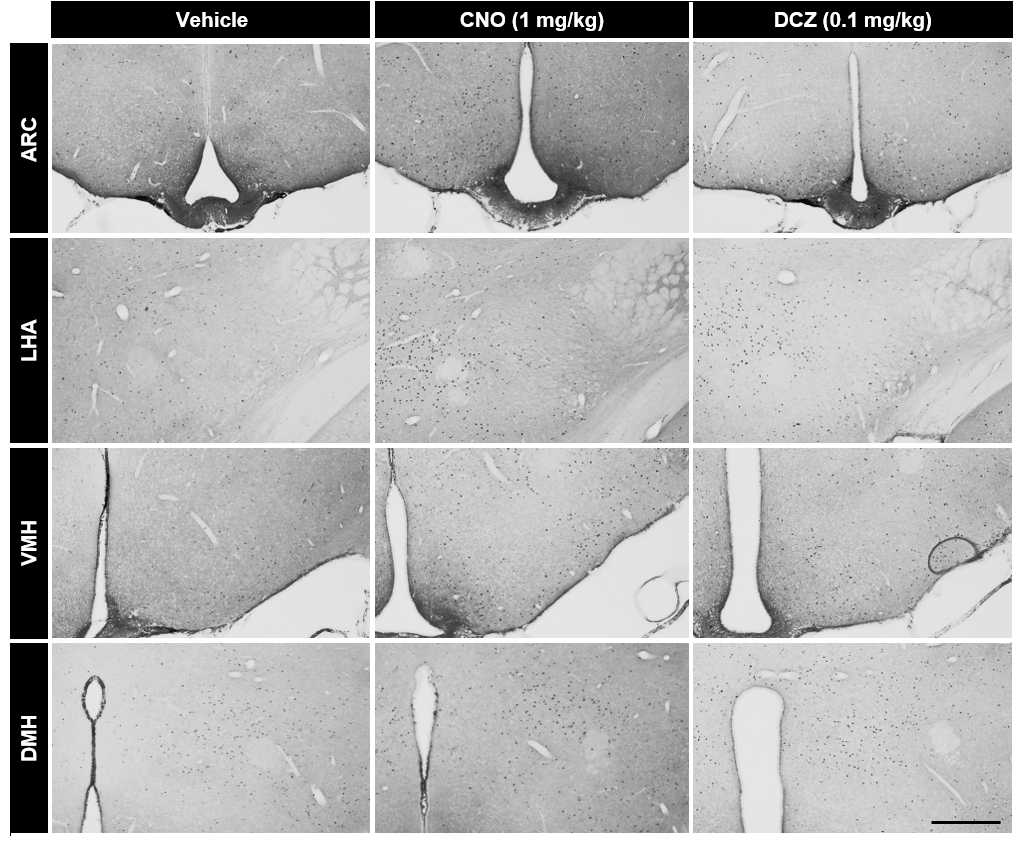


**Supplementary Figure 4. Representative images of DAB immunostaining for Fos in the arcuate nucleus (ARC), lateral hypothalamic area (LHA), ventromedial hypothalamus (VMH), and dorsomedial hypothalamus (DMH).**

The images are obtained at 120 min after s.c. injections of vehicle, CNO, or DCZ. Fos-positive neurons are stained by dark brown. Images are converted to black and white to improve visibility. Scale bar, 400 µm.


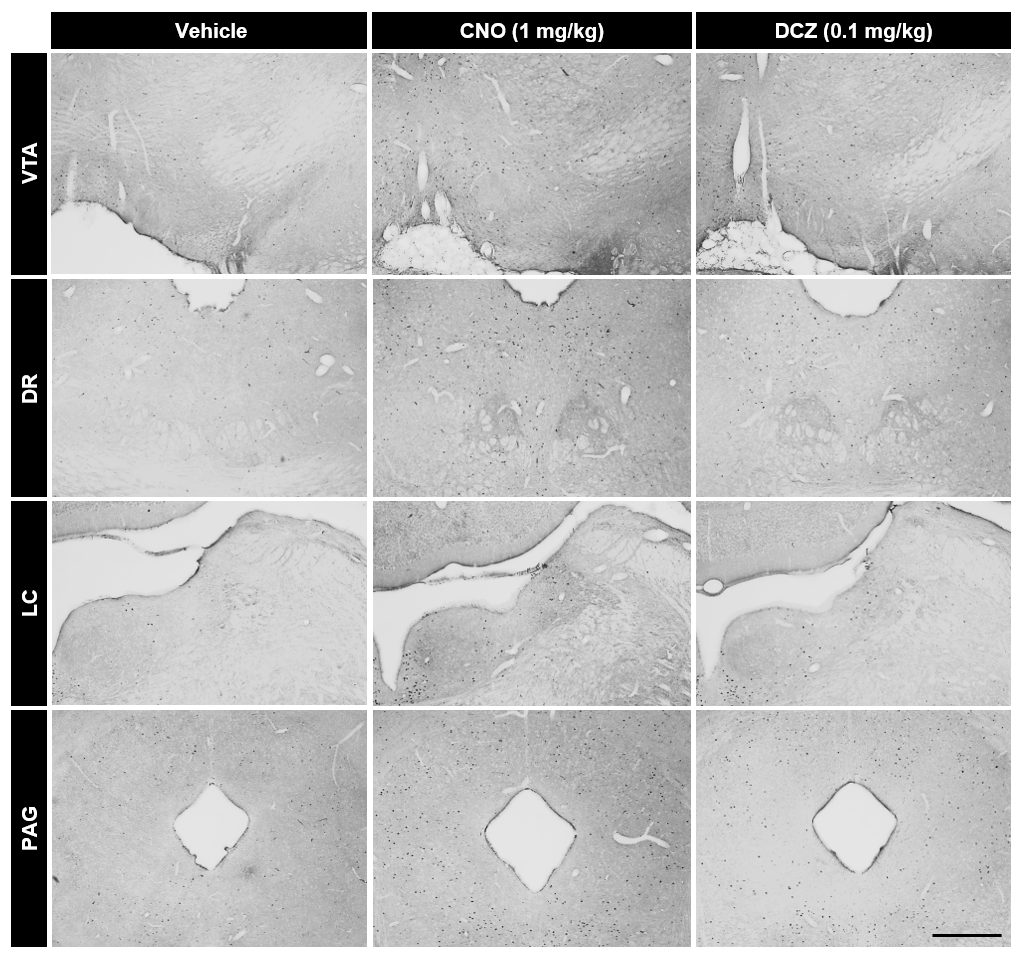


**Supplementary Figure 5. Representative images of DAB immunostaining for Fos in the ventral tegmental area (VTA), dorsal raphe nucleus (DR), locus coeruleus (LC), and periaqueductal gray (PAG).**

The images are obtained at 120 min after s.c. injections of vehicle, CNO, or DCZ. Fos-positive neurons are stained by dark brown. Images are converted to black and white to improve visibility. Scale bar, 400 µm.


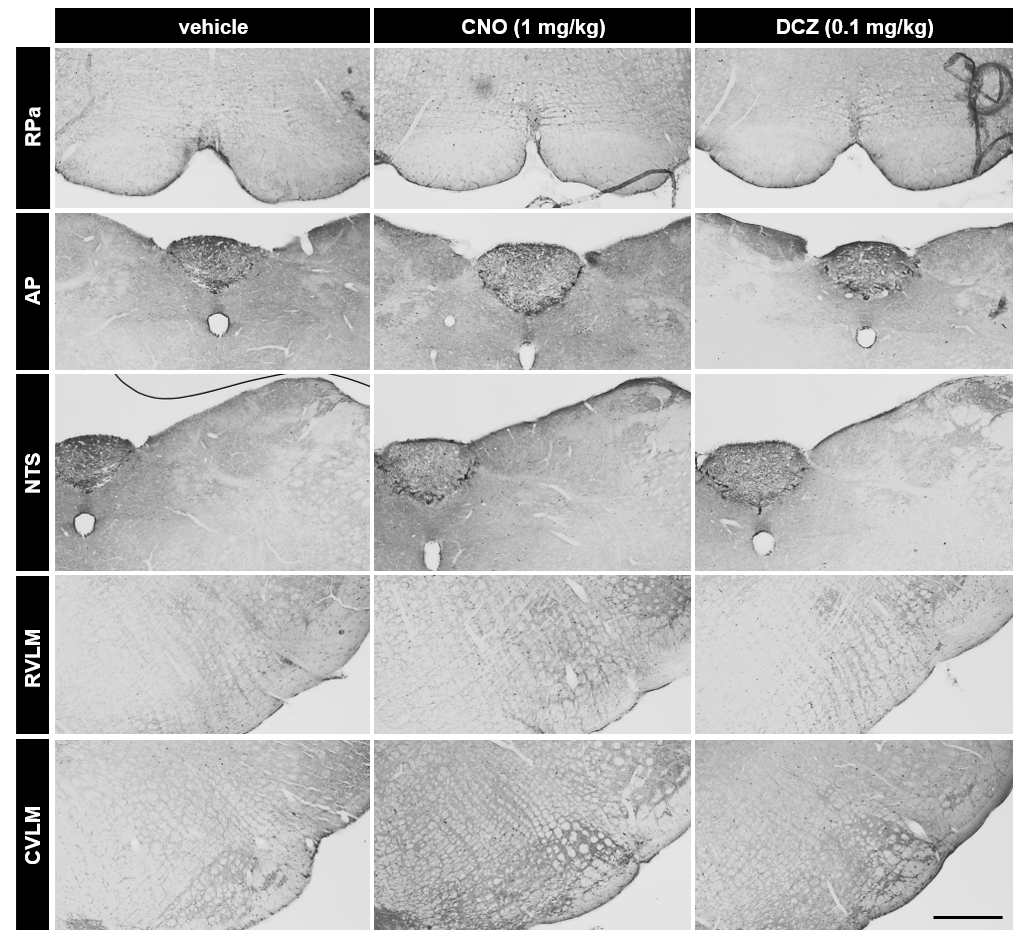


**Supplementary Figure 6. Representative images of DAB immunostaining for Fos in the brainstem nuclei.**

The images are obtained at 120 min after s.c. injections of vehicle, CNO, or DCZ. Fos-positive neurons are stained by dark brown. Images are converted to black and white to improve visibility. Scale bar, 400 µm. RPa, raphe pallidus; AP, area postrema; NTS, nucleus tractus solitarius; RVLM, rostral ventrolateral medulla; CVLM, caudal ventrolateral medulla.
